# YeastSpotter: accurate and parameter-free web segmentation for microscopy images of yeast cells

**DOI:** 10.1093/bioinformatics/btz402

**Published:** 2019-05-16

**Authors:** Alex X Lu, Taraneh Zarin, Ian S Hsu, Alan M Moses

**Affiliations:** 1 Department of Computer Science, University of Toronto, Toronto, ON, Canada; 2 Department of Cells and Systems Biology, University of Toronto, Toronto, ON, Canada; 3 Center for Analysis of Genome Evolution and Function, University of Toronto, Toronto, ON, Canada

## Abstract

**Summary:**

We introduce YeastSpotter, a web application for the segmentation of yeast microscopy images into single cells. YeastSpotter is user-friendly and generalizable, reducing the computational expertise required for this critical preprocessing step in many image analysis pipelines.

**Availability and implementation:**

YeastSpotter is available at http://yeastspotter.csb.utoronto.ca/. Code is available at https://github.com/alexxijielu/yeast_segmentation.

**Supplementary information:**

Supplementary data are available at *Bioinformatics* online.

## 1 Introduction

The accurate segmentation of a microscopy image into single cells is an important preprocessing step for many image analysis pipelines (Meijering, 2012). As a model organism, the budding yeast *Saccharomyces cerevisiae* is often used in imaging experiments, some of which can generate tens of thousands of images (Dubreuil *et al.*, 2018; Koh *et al.*, 2015; Riffle and Davis, 2010; Weill *et al.*, 2018). To analyze these images, a range of segmentation options have emerged, often tailored to specific datasets. Some integrate assumptions specialized to screens, such as the presence of fluorescent markers (Handfield *et al.*, 2013), edge patterns (Dimopoulos *et al.*, 2014; Wang *et al.*, 2018), or assumptions specific to microfluidics experiments (Bakker *et al.*, 2018). Others require the laborious specification of many manual parameters (Carpenter *et al.*, 2006); indeed, most methods for brightfield images require extensive parameter tuning for optimal performance (Versari *et al.*, 2017).

For a cell biologist, the wide choice and complexity of segmentation methods may lead them to manual quantification if the effort required for automation appears disproportionate to the scale of their experiments. We envisioned a tool that could produce reasonable segmentations for most images with minimal effort. Toward this goal, we designed YeastSpotter (yeastspotter.csb.utoronto.ca), a web application that generalizes to images from different microscopes and imaging modalities, without the need to specify any parameters: the user simply submits their images and obtains a segmentation. Despite its simple use, we obtain comparable performance to specialized state-of-the-art methods on benchmarks for segmentation of both fluorescent and brightfield images.

## 2 Materials and methods

Our underlying segmentation method is based upon transferring publicly available convolutional neural networks from the 2018 Kaggle Data Science Bowl competition. In this competition, contestants trained models to segment images of mostly human nuclei, using image set BBBC038v1 from the Broad Bioimage Benchmark Collection (Ljosa *et al.*, 2013) as training data. Despite not being trained on yeast cells, we found that these models transferred well without fine-tuning. We used a pre-trained mask-RCNN model (He *et al.*, 2017) by the third-place winner, the Deep Retina team, which we chose due to its simplicity and easily extensible code. To make this model more accessible to the community, we implemented YeastSpotter as a web application to run images through this model.

To use YeastSpotter, the user simply uploads their image, which redirects them to a page that tracks the progress of their request and produces segmentation results once ready. A preview image on the result page shows the outlines of the segmentation overlaid on the original input. The user can then download the segmentation, which is stored as an integer-signed tiff file (pixels with a value of 0 correspond to the background, while pixels belonging to each unique cell are each assigned a different integer value). On the website, we provide instructions for loading these fines into ImageJ and scripts to read them in Python, Matlab and R.

YeastSpotter is intended for low-throughput use and only accepts a single image per request. For batch segmentation, we also provide user-friendly Python code (www.github.com/alexxijielu/yeast_segmentation).

## 3 Results

To understand the accuracy and run-time of the segmentations produced by our method, we used a set of 4305 ellipses manually drawn around yeast cells in fluorescent micrographs (Handfield *et al.*, 2013). We compared segmentations from YeastSpotter to previously reported results for segmentation software specially designed for this dataset by Handfield *et al.* (Handfield *et al.*, 2013), and for segmentations obtained through CellProfiler (Carpenter *et al.*, 2006) in Table 1, using parameters previously optimized by Chong *et al.* (Chong *et al.*, 2015). These results suggest that our method segments fluorescent micrographs of yeast cells more accurately than established methods, with no manual tuning of parameters.

**Table 1.** Benchmark results on fluorescent yeast micrographs

Method	Ellipses matched	Mean	Standard deviation	Correlation	Run time
YeastSpotter	97.5%	1.58	0.99	0.969	1172
Handfield *et al.* (2013)	92.3%	1.41	1.21	0.928	13 851
CellProfiler	89.0%	2.23	1.80	0.876	231

We report the percent of manual ellipses with a matched single-cell segmentation within ten pixels, the mean and standard deviation of distance (in pixels) between the centers of the manual ellipse and segmentation, the correlation between their areas and the time (in seconds) to process the evaluation image set (68 images).

To test the generalization capacity, we evaluated our segmentations on detecting cell centers in brightfield images from the Yeast Image Toolkit benchmark (Versari *et al.*, 2017, Supplementary Fig. S1). We achieved comparable performance to most tools, even though they have been extensively optimized for brightfield images (Versari *et al.*, 2017), while YeastSpotter was not. We note that YeastSpotter does not achieve state-of-the-art performance, so expert users may still want to optimize tools for their images.

**Fig. 1.**
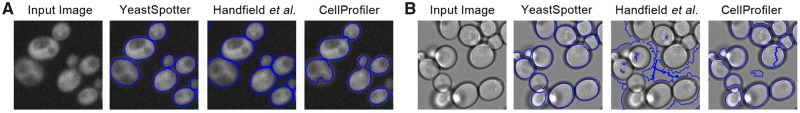
Qualitative segmentation results for various segmentation algorithms. We show results for fluorescent (**A**) and brightfield (**B**) images. In the left-most panels, we show the original input image. In the other panels, we show outlines of the segmentation result from each segmentation method (as labeled) overlaid on the original image in blue

We next qualitatively examined segmentation results on these (Fig. 1) and other image modalities (Supplementary Fig. S2 shows differential interference contrast (DIC) and phase contrast). On fluorescent images (Fig. 1A), CellProfiler (with parameters used for Table 1) under-segments bud cells, grouping the pixels of bud cells with mother cells and does not accurately detect the boundaries of cells with dim vacuoles. Accurately segmenting bud cells is critical for understanding yeast biology, as it permits for the study of the cell-cycle (Handfield *et al.*, 2013). YeastSpotter and the method of Handfield *et al.* more reliably separate bud cells from mother cells.

However, as the method of Handfield *et al.* is engineered for fluorescent images, it fails to generalize to the segmentation of brightfield images (Fig. 1B). The CellProfiler segmentation optimized for fluorescent micrographs is more robust, but still produces many errors, identifying parts of the background as cells and over-segmenting some cells. YeastSpotter performs well on both fluorescent and brightfield images; there are some errors with overlapping or out-of-focus cells in the brightfield images, but most cells are segmented well.

## 4 Conclusion

Here, we introduced a user-friendly and generalizable web application for the segmentation of yeast microscopy images. We produced high-quality segmentations for both fluorescent and brightfield images using the same model and parameters. These results suggest that YeastSpotter is highly general, as opposed to most previous methods, which have been developed to segment images of a particular type.

YeastSpotter may not outperform carefully optimized methods tailored to specific problems. However, for users without the time or expertise to fine-tune or compare specialized methods, our method offers excellent off-the-shelf performance.

## Supplementary Material

btz402_Supplementary_Data
