# Supplementary material for: YeastSpotter: accurate and parameter-free web segmentation for microscopy images of yeast cells
Source: Bioinformatics. 2019 May 16;35(21):4525–7. doi: 10.1093/bioinformatics/btz402 (PMC6821424; doi:10.1093/bioinformatics/btz402)
Supplement: btz402_Supplementary_Data [file btz402_supplementary_data.docx]

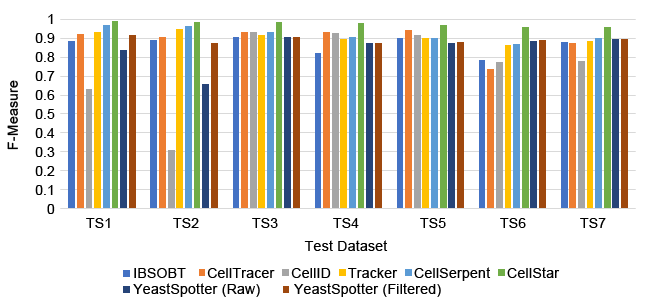


**Supplementary Figure 1.** F-measures for cell detection performance on brightfield test sets in the Yeast Image Toolkit. As a general tool, YeastSpotter is sensitive to dust particles in the noisier test sets (TS1 and TS2), but these are easily removed with a trivial filter by object size with negligible loss of real cells. Thus, we report results for both raw segmentations from YeastSpotter and where all objects under 150 pixels have been removed. For all other tools, we list previously reported results from Versari *et al*.


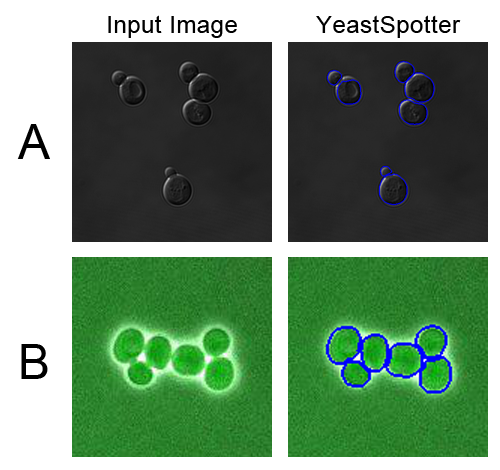


**Supplementary Figure 2.** YeastSpotter segmentation results for differential interference contrast (A) and phase contrast (B) microscopy images. Phase contrast images were taken from the Cell Image Library, contributed by Ball *et al*. (PLoS One 2011).
